# Flavin dependency undermines proteome stability, lipid metabolism and cellular proliferation during vitamin B2 deficiency

**DOI:** 10.1038/s41419-020-02929-5

**Published:** 2020-09-07

**Authors:** Adrían Martínez-Limón, Giulia Calloni, Robert Ernst, R. Martin Vabulas

**Affiliations:** 1 Buchmann Institute for Molecular Life Sciences, Goethe University Frankfurt, Frankfurt am Main, Germany; 2 Institute of Biophysical Chemistry, Goethe University Frankfurt, Frankfurt am Main, Germany; 3 Center for Molecular Signaling (PZMS), Institute of Medical Biochemistry and Molecular Biology, Medical Faculty, University of Saarland, Homburg, Germany; 4 Present Address: Institute for Research in Biomedicine (IRB), Barcelona, Spain; 5 Present Address: AB SCIEX Germany GmbH, Darmstadt, Germany; 6 Present Address: Charité – Universitätsmedizin Berlin, Institute of Biochemistry, Berlin, Germany

**Keywords:** Enzymes, Lipidomics, Protein folding

## Abstract

Tumor cells adapt their metabolism to meet the energetic and anabolic requirements of high proliferation and invasiveness. The metabolic addiction has motivated the development of therapies directed at individual biochemical nodes. However, currently there are few possibilities to target multiple enzymes in tumors simultaneously. Flavin-containing enzymes, ca. 100 proteins in humans, execute key biotransformations in mammalian cells. To expose metabolic addiction, we inactivated a substantial fraction of the flavoproteome in melanoma cells by restricting the supply of the FMN and FAD precursor riboflavin, the vitamin B2. Vitamin B2 deficiency affected stability of many polypeptides and thus resembled the chaperone HSP90 inhibition, the paradigmatic multiple-target approach. In support of this analogy, flavin-depleted proteins increasingly associated with a number of proteostasis network components, as identified by the mass spectrometry analysis of the FAD-free NQO1 aggregates. Proteome-wide analysis of the riboflavin-starved cells revealed a profound inactivation of the mevalonate pathway of cholesterol synthesis, which underlines the manifold cellular vulnerability created by the flavoproteome inactivation. Cell cycle-arrested tumor cells became highly sensitive to alkylating chemotherapy. Our data suggest that the flavoproteome is well suited to design synthetic lethality protocols combining proteostasis manipulation and metabolic reprogramming.

## Introduction

The uncontrollable proliferation and invasiveness of malignantly transformed cells are related with high energetic and anabolic costs. To meet these needs, tumor cells reprogram the metabolism resulting in transformation-specific enzymatic patterns. Actually, one of the distinct metabolic features of tumors, the aerobic glycolysis, was noticed long before the dawn of the oncogene-centric understanding of the tumorigenesis^1^. It is well established now that cancer cells deregulate the uptake of glucose and amino acids, activate alternative modes of nutrient acquisition, use glycolysis and citric cycle intermediates for anabolism among many other changes in addition to the originally noticed increase of lactate production^2^. These changes have inspired the development of numerous therapeutic strategies^3,4^. For example, 2-deoxyglucose was used to inhibit hexokinase and slow down the uptake of glucose, arginine deiminase was applied to exploit arginine-dependence of some tumors, or, recently, glutaminase inhibitors were tested as a means to target the upregulated glutaminolysis in cancers.

Unfortunately, metabolic as well as other therapeutic schemes in oncology face two difficulties inherent to tumors, namely, the heterogeneity and adaptability of transformed cells^5^. Consequentially, combination of different substances has been proposed early on and used since in a hope of targeting different tumor sublcones and restricting their evolution more efficiently. The enthusiasm regarding the combinatorial strategy is supported by the fact that tumors develop a number of functional liabilities, called non-oncogene addiction, as a consequence of the tumorigenic processes^6^. For example, the rate of DNA damage and the severity of replication stress are enhanced, and transformed cells try to ameliorate this stress by activating the DNA damage response^7^. Similarly, the spindle checkpoint helps tumor cells cope with the defects in the mitotic machinery and overcome the mitotic stress^8^. Another notorious example of non-oncogene addiction is the activation of the HSF-dependent proteostasis machinery which rescues mutant oncoproteins from degradation^9^. Inhibition of these and other protective reactions has been tested as a part of combination therapies aiming to induce synthetic lethality, which has resulted in varying success^10^.

While looking for novel therapeutic possibilities to increase metabolic stress in tumors, we considered the group of flavin-containing enzymes, ca. 100 proteins in the human proteome. Flavoproteins are responsible for a number of key biotransformations and their mutations result in severe diseases^11^. Therefore, we propose here the inactivation of the entire flavoproteome as a means to increase the vulnerability of tumor cells. The synthetic lethality approach was tested by restricting the supply of the FMN and FAD precursor riboflavin, the vitamin B2, in cell culture of the melanoma cell line B16. In addition to enzymatic defects, riboflavin deficiency interferes with the final structural maturation of a set of polypeptides^12^. This latter effect resembles mechanistically the chaperone HSP90 inhibition, the paradigmatic multiple-target therapeutic approach to exploit proteostasis stress and stress response-dependence of tumorigenesis^13^. Simultaneous targeting of key tumor cell functions, such as mitochondrial ATP production, cholesterol biosynthesis and cell proliferation, through flavoproteome destabilization deserves a thorough biochemical and clinical evaluation.

## Materials and methods

### Cell culture, transfection, and immunoblotting

B16 (subclone F0) murine melanoma cell line (CRL-6322, ATCC, USA) was cultured in Dulbecco’s modified Eagle’s medium (DMEM) supplemented with 10% fetal bovine serum (FBS), 2mM l-glutamine, 100 IU/ml penicillin G, 100 μg/ml streptomycin sulfate and non-essential amino acids (Gibco, Germany). Riboflavin-deficient medium was prepared omitting riboflavin from the usual DMEM mix. After pH adjustment to 7.5 and filtration, medium was stored for several weeks at 4 °C. Before using, medium was supplemented with 10% dialyzed FBS (Thermo Scientific, USA) and 1 µM riboflavin as needed for comparison.

B16 cells were transfected by electroporation in 400 μl intracellular buffer (135 mM KCl, 0.2 mM CaCl_2_, 2 mM MgCl_2_, 5 mM EGTA, 10 mM HEPES pH 7.5) freshly supplemented with 25% FBS at 250 V and 950 µF using GenePulser Xcell (Bio-Rad Laboratories, USA). After electroporation, cells were washed and recovered in normal medium for 4–5 h. Riboflavin deprivation and inhibitors as needed were then applied for 24 h.

For immunoblotting, cells were harvested by trypsinization and lysed in lysis buffer (20 mM HEPES pH 7.5, 0.5 % IGEPAL CA-630, 100 mM KCl, 10 mM MgCl_2_ and 10% Glycerol). In the case of phosphorylation-specific analysis, phosphatese inhibitor cocktail (Sigma-Aldrich, USA) was added to the lysis buffer. After adding reducing SDS sample buffer, lysates were resolved using 10% SDS-PAGE and transferred onto blotting membranes. Membranes were blocked with 5% skim milk solution, probed with the indicated antibodies and developed using the ECL Prime Western Blotting Detection Reagent (GE Healthcare, UK).

### ATP measurements

ATP quantification was carried out using ATP assay kit MAK190 (Sigma-Aldrich) according to the manufacturer’s instructions. Briefly, cells were incubated in riboflavin-deficient medium for three days, then harvested and lysed in ATP assay buffer provided with the kit. Samples were normalized using Bradford reagent and deproteinized using a Microcon-30kDa Centrifugal Filter Unit. Fluorescence was excited at 535 nm and recorded at 587 nm in 96-well plates.

### HSP70 Induction

B16 cells cultured for two days in normal or riboflavin deficient medium were incubated for 1 h at 45 °C and then transferred back for the recovery at 37 °C for 5 h. Cells were harvested, washed twice in PBS, and lysed in 200 µL of CHIP lysis buffer (20 mM HEPES pH 7.5, KOH, 0.5% IGEPAL CA-630, 100 mM KCl, 10 mM MgCl_2_, and 10% glycerol). SDS sample buffer was added to normalized samples, samples were heated for 5 min at 95 °C and analysed by western blotting.

### Translation rate analysis

To assess the translation rate of B16 cells under different conditions, cells were transfected with 30 µg of Ubiquitin-eGFP expression plasmid by means of electroporation and plated on 10 cm dishes. After 6 h, the medium was exchanged to fresh normal or riboflavin deficient and cells were incubated for 2 days. On the second day, cells were treated with 50 µM MG132 for 5 h, harvested, washed twice with PBS, and lysed in CHIP lysis buffer. SDS sample buffer was added to normalized samples, samples were heated for 5 min at 95 °C and analysed by western blotting.

### MMS and mitomycin C toxicity

Cellular toxicity assays were performed using either methyl methanesulfonate (MMS) or Mitomycin C (both from Sigma-Aldrich). Cells were plated on 6-well plates and incubated for 3 days in normal or riboflavin-free medium. On the third day, either 500 µM MMS or 100 µM Mitomycin C were added for additional 24 h in the same medium. Released and attached cells were then collected, pelleted, directly resuspended in a staining solution (Complete DMEM with 30 nM SYTOX™ Green Dead Cell Stain from Thermo Fisher) and incubated for 15 min in the dark. S3 Cell Sorter was used to quantify dead cells. Approximately 20.000 cells were acquired per sample. The data obtained were analysed using FCS Express 5 Flow package (De Novo Software, USA).

### BrdU proliferation assay

B16 cells were plated on poly-L-lysine-coated cover glasses in 12-well plates. Cells were cultured for 3 days in normal or riboflavin-deficient medium and 10 µM BrdU (Merck Millipore, Germany) pulse was carried out for 5 h. After extensive washing with PBS, cells were fixed with 4% paraformaldehyde solution for 45 min at room temperature and then incubated with 0.1% Triton X-100 solution for 20 min at room temperature for permeabilization. To denature DNA, 1.5 M HCl solution was added to fixed cells for 30 min at room temperature. Cells were washed three times with PBS, blocked with 3% BSA/PBS solution for 30 min at room temperature and incubated with AlexaFluor^®^ 488-conjugated anti-BrdU antibody (Merck-Millipore) O/N at 4 °C. To stain DNA, DAPI solution (1:5000) was added for 3 min at room temperature. Microscopy cover glasses were mounted with PBS. Cells were imaged using a Zeiss LSM 780 confocal microscope (Germany).

### Cell cycle analysis

Cells were plated on 10-cm dishes, incubated for 3 days in normal or riboflavin-deficient medium, harvested by trypsinization, washed with ice-cold PBS twice and fixed using 100% ice-cold ethanol. Fixation was carried out by adding ethanol drop-wise on top of a 300 µL cell suspension in PBS while vortexing very softly. Fixed cells were incubated O/N at 4 °C, washed with PBS twice and resuspended in propidium iodide staining solution (50 µg/mL propidium iodide and 100 µg/mL RNase A (both from Sigma-Aldrich) in PBS) for 30 min at room temperature. Cells were vortexed gently every 10 min to avoid their excessive sedimentation. S3 Cell Sorter (Bio-Rad Laboratories) was used to analyze approximately 20.000 cells per sample. FCS Express 5 (De Novo Software) was used to analyze the data. The Multi-Cycle DNA analysis tool in FCS Express 5 was used to quantify cell cycle profiles.

### β-Galactosidase assay

B16 cells were plated on 6-well plates and incubated for 3 days in normal or riboflavin-deficient medium. Cells were washed with PBS twice and fixed with freshly prepared 3.7% formaldehyde/PBS solution for 5 min at room temperature. Fixed cells were incubated with X-gal staining solution (40 mM citric acid/sodium phosphate pH 4.0, 1 mg/mL X-gal, 5 mM potassium ferricyanide, 5 mM potassium ferrocyanide, 150 mM NaCl, and 2 mM MgCl_2_). Staining buffer was prepared immediately before use. Cells were incubated overnight at 37 °C and normal CO_2_ concentration. Next day, staining solution was removed and replace with water. Images were taken immediately using a Leica MC170 HD camera attached to an inverted light microscope with a 5x magnification objective.

### Localization of farnesylated EGFP

Plasmid for mammalian expression of EGFP fused to the C-terminal 20 amino acid farnesylation signal of HRas^14^ was used to prepare B16 stably transfected cell line. For imaging, cells were plated on 24-well black plates with clear film bottom (Eppendorf, Germany) and incubated for 3 days in normal or riboflavin-free medium. Zeiss LSM 780 confocal microscope was used to obtain fluorescent pictures of living cells at 63x magnification with immersion oil. The images were analysed using ImageJ.

For additional information see “SI Materials and Methods”.

## Results

### Depletion of riboflavin results in coaggregation of apoprotein NQO1 with bystander proteins

**Fig. 1:**
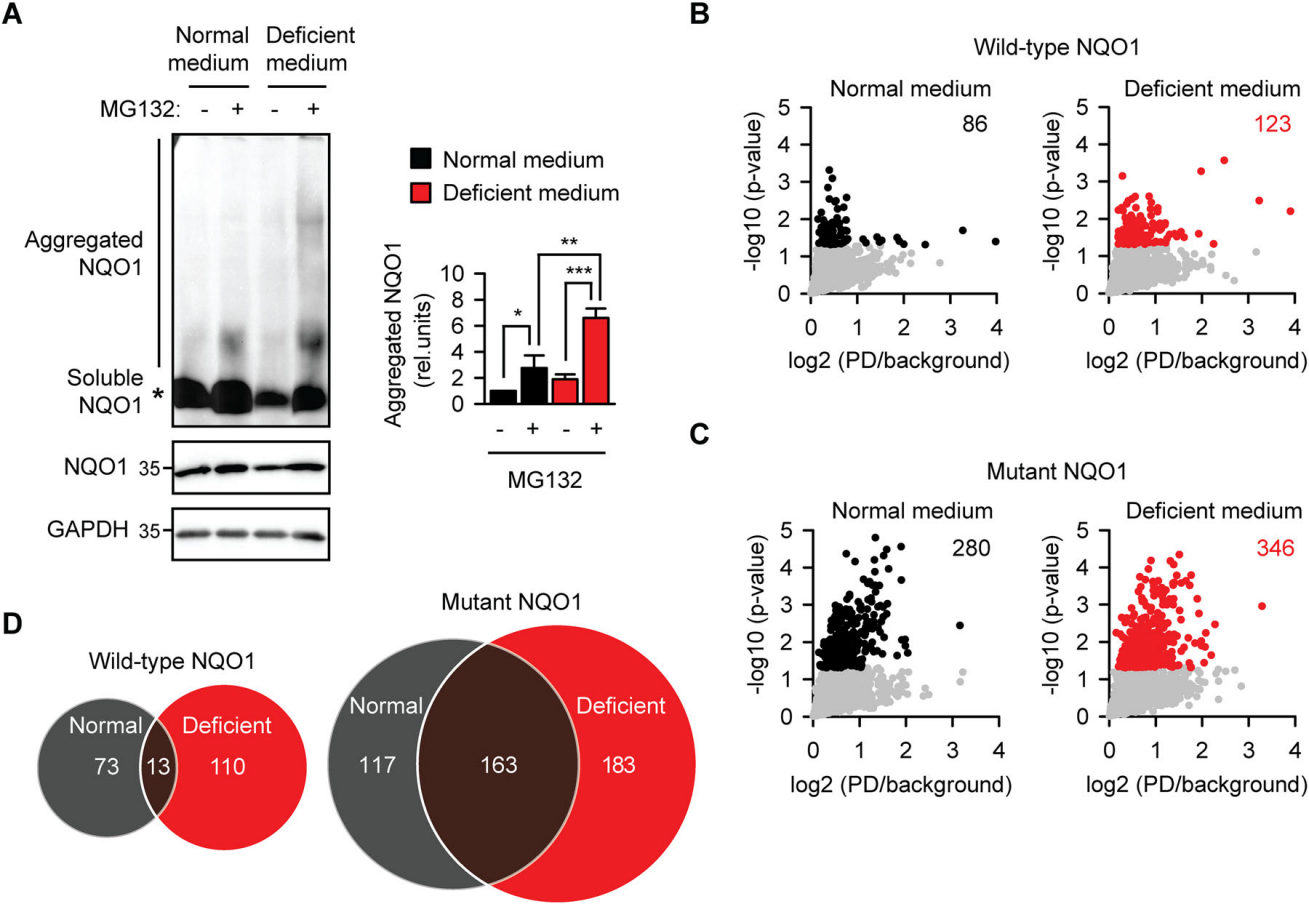
Depletion of riboflavin leads to coaggregation of apoprotein NQO1 with bystander proteins in melanoma cells. **a** FLAG-NQO1-transfected B16 murine melanoma cells were incubated in normal or riboflavin-deficient medium for two days. Proteasomal degradation was inhibited with 10 μM MG132 for the last 16 h as indicated. Lysates were prepared and aggregated NQO1 was quantified using blue native gel electrophoresis and anti-FLAG western blotting (upper gel). The total amount of NQO1 was determined by western blotting after SDS-PAGE (middle gel). GAPDH was used as loading control. *p < 0.05, **p < 0.01, ***p < 0.001, two-tailed *t* test (*N* = 3, mean ± SD). **b**, **c** Wild-type or P187S mutant FLAG-NQO1-transfected B16 murine melanoma cells were incubated in normal or riboflavin-deficient medium for two days. Proteasomal degradation was inhibited with 10 μM MG132 for the last 16 h. FLAG-NQO1 pulldowns were prepared as detailed in Materials and Methods and label-free mass spectrometry was used to quantitate proteins associated with NQO1(*N* = 4 biological replicates). Volcano plots indicate average enrichment of individual protein levels. Red and black colors mark significantly enriched proteins as determined by two-sample *t* test (*p* < 0.05), the numbers are explicitly indicated on the respective panels. **d** Overlap of interactors with wild-type and mutant NQO1 in normal and riboflavin-deficient medium.

In crowded cellular environment, misfolded or otherwise destabilized proteins have propensity for aberrant interaction with other proteins. Reciprocally, the number and extent of non-natural associations can be used as an indication of the destabilization of a protein. The aggregates can be visualized as a high molecular weight smear during native gel electrophoresis. Recombinant cofactor-free NQO1 (apo-NQO1) coaggregates with Aβ1–42 amyloid in vitro^12^. Using native gel electrophoresis of cellular lysates, we could detect a fraction of NQO1 as higher molecular weight species under riboflavin starvation for two days (Fig. 1a). Inhibition of proteasome with MG-132 was needed to prevent the degradation of apo-NQO1. The NQO1 aggregation was reversible because the addition of the cofactor FAD to the lysate significantly reduced the amount of the higher molecular weight species (Fig. S1A). This effect was highly specific as indicated by the lack of an effect upon addition of the FAD precursor riboflavin.

Bystander coaggregation can deplete cells of functional proteins and thus contribute to the proteotoxicity^15^. We set out to determine if this scenario happens during vitamin B2 starvation. Using label-free quantitative mass spectrometry, we identified proteins associated with wild-type NQO1 in melanoma cells under normal or riboflavin-deficient conditions (Dataset S1-S3). In parallel, we used P187S mutant of NQO1 for comparison because this mutant and the wild-type apo-NQO1 are processed similarly by the protein quality control machinery^12^. In support to the bystander coaggregation, more NQO1-associated proteins were detected under riboflavin deficiency (Fig. 1b). Mutant NQO1 is known to be unstable even under normal conditions, accordingly, its interactome was substantially bigger and increased further in riboflavin-deficient medium (Fig. 1c). Poor overlap between the wild-type NQO1 interactomes in normal and deficient conditions supported the aberrancy of associations during riboflavin starvation (Fig. 1d). On the other side, the overlap between the mutant NQO1 interactomes in different conditions was higher, which indicated the bystander coaggregation with the mutant protein even during riboflavin sufficiency.

### NQO1 aggregates associate with molecular chaperones

**Fig. 2:**
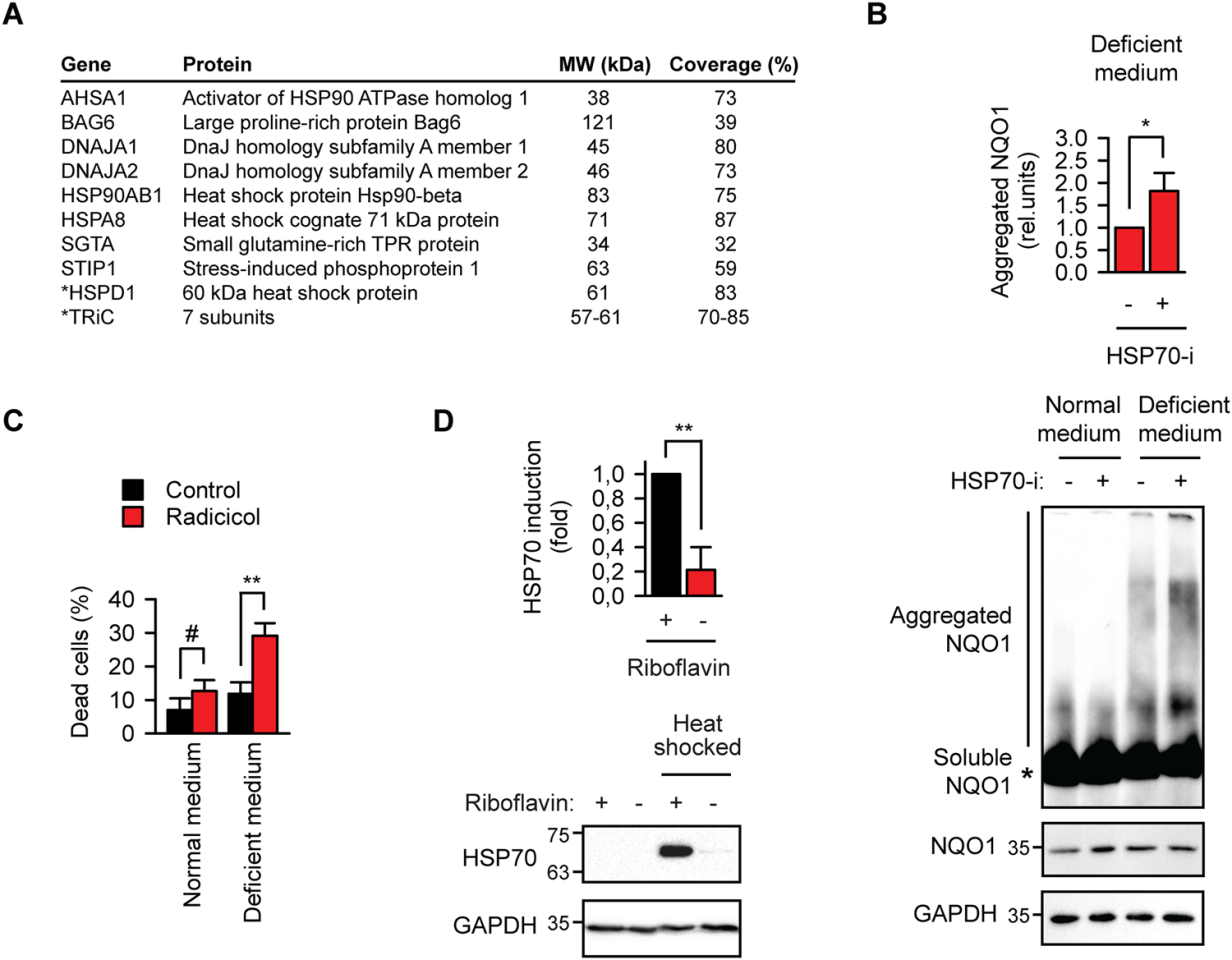
NQO1 aggregates associate with molecular chaperones. **a** Molecular chaperones significantly associated with wild-type apo-NQO1 and mutant NQO1 as determined by mass spectrometry analysis (*N* = 4, two-sample *t* test, *p* < 0.05). Molecular weight (MW) and sequence coverage by MS measurements are indicated. *, chaperones significantly enriched also in wild-type NQO1 complexes under riboflavin-sufficient conditions. **b** FLAG-NQO1-transfected B16 murine melanoma cells were incubated in normal or riboflavin-deficient medium for two days. For the last 16 h, 10 μM MG132 was added for all samples and HSP70 inhibitor VER-155008 was added at 10 μM as indicated. Lysates were prepared and aggregated NQO1 was quantified using blue native gel electrophoresis followed by anti-FLAG western blotting (upper gel). The total amount of NQO1 was determined by western blotting after SDS-PAGE (middle gel). GAPDH was used as loading control. *p < 0.05, two-tailed *t* test (*N* = 3, mean ± SD). **c** Cell death in normal or riboflavin-deficient medium upon 24 h incubation with HSP90 inhibitor radicicol as determined with SYTOX dye and flow cytometric quantification. #, not significant difference, **p < 0.01, two-tailed *t* test (*N* = 3, mean ± SD). **d** HSP70 induction upon 1 h 45 °C heat shock is affected by riboflavin deficiency. Cells were analyzed upon 5 h recovery at 37 °C. GAPDH was used as loading control. **p < 0.01, two-tailed *t* test (*N* = 3, mean ± SD).

High overlap between normal and mutant NQO1 interactomes in deficient medium underscores common structural determinants of associations under these conditions (Fig. S1B). Consistently, when the intersection of all three aberrant interactomes (Fig. S2A) was analyzed, an enrichment of molecular chaperones was observed (Fig. 2a). HSP70 and HSP90 chaperones together with their cofactors were present in NQO1 aggregates and absent from wild-type NQO1 interactors in normal medium. Inhibition of the HSP70 or HSP90 activity enhanced the formation of NQO1 aggregates or the cytotoxicity in riboflavin-deficient medium, respectively (Fig. 2b, c). HSP70 induction by acute proteostasis stress was also affected in deficient medium (Fig. 2d), which cannot be attributed to protein translation differences (Fig. S2B). Activation of AMPK during metabolic stress is known to inhibit transcription factor HSF1 and thus is one possibility of the lacking HSP70 induction in starving cells.

In summary, these data indicated that acute deficit of vitamin B2 led to the coaggregation of the cofactor-free flavoprotein with bystander polypeptides and its extensive association with molecular chaperones.

### Riboflavin starvation leads to metabolic reprogramming of melanoma cells

**Fig. 3:**
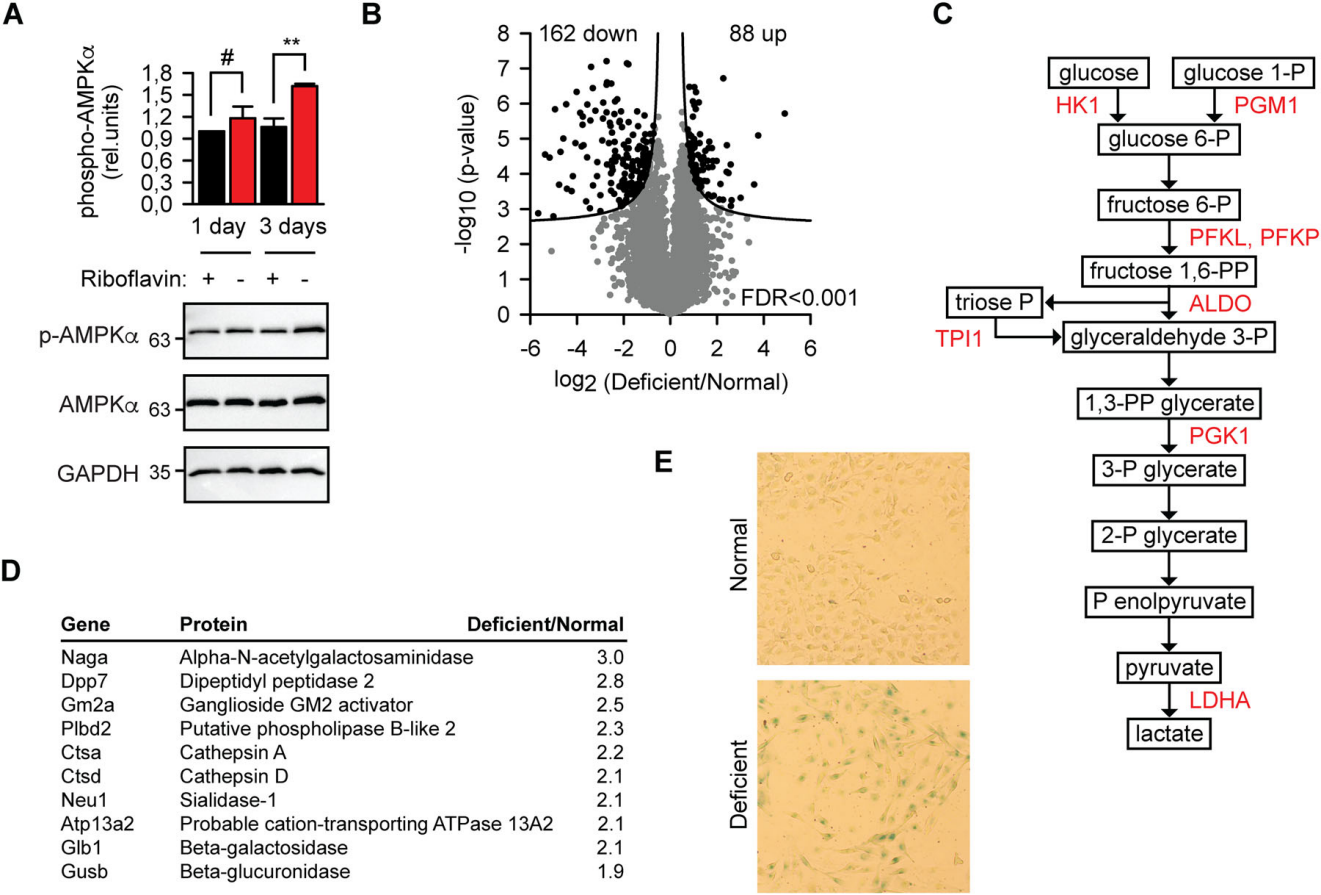
Riboflavin starvation results in metabolic reprogramming of melanoma cells. **a** Catalytic subunit α of AMPK is activated by the Thr172 phosphorylation as detected by the phoshospecific antibody (p-AMPKα) in B16 lysates after 3 days starvation in riboflavin-free medium. GAPDH was used as loading control. #, not significant difference, **p < 0.01, two-tailed *t* test (*N* = 3, mean ± SD). **b** Volcano plot of quantified proteins plotted according to their change in riboflavin-deficient medium for 3 days in B16 cells. The statistical significance of the respective ratios is plotted on the y-axis. False discovery rate (FDR) used to define the upregulated and downregulated proteins (black symbols) is indicated. *N* = 4 independent experiments. **c** Glycolysis scheme with indicated enzymes which were significantly upregulated during 3 days culture in riboflavin-deficient medium. **d** A list of lysosomal proteins which were upregulated during 3 days culture in riboflavin-deficient medium as determined by mass spectrometry analysis of B16 lysates. Average change is indicated (*N* = 4). **e** Histochemichal staining to detect lysosomal galactosidase activity in B16 melanoma cells after 3 days starvation in riboflavin-free medium. Representative bright-field view images from one out of three independent experiments are shown.

Flavoproteins are key components of the mitochondrial respiratory chain complexes, thus riboflavin deficiency is expected to result in AMPK activation via an elevated AMP/ATP ratio. However, AMPK was activated weakly and only with delayed kinetics (Fig. 3a). Weak activation of AMPK correlated with modest reduction of ATP levels (Fig. S3A). One reason why cells managed to keep quite high ATP concentration could be their metabolic reorganization under riboflavin starvation. This assumption was investigated in the next step. In addition to phosphorylation-related activity changes of target polypeptides, protein level variation represents another way how cells adjust to changing environment. An in-depth analysis of protein levels in riboflavin-starved cells revealed a distinct reorganization of the proteome (Fig. 3b). Among the significantly downregulated clusters, one related to electron transport and ATP synthesis processes (Fig. S3B). This is a non-trivial observation because it underscores the structural, not merely functional, relevance of flavin cofactors for the respective enzymes. At least some flavoproteins seem to be destabilized and their levels decrease when flavin cofactor is missing. The damage was not unspecific to all metabolic processes. In parallel to the downregulation of mitochondrial respiration, the glycolysis-related cluster became upregulated (Fig. S3C). Actually, the set of GO Biological Process categories related to glycolysis was the only one significantly enriched among upregulated proteins upon 3 days of riboflavin starvation. With 8 upregulated enzymes, the metabolic reprogramming towards glycolysis turned out to be comprehensive and manifold (Fig. 3c).

The second noticeable metabolic reorganization took place at the lysosome. At least nine lysosomal hydrolases became upregulated more than two times in riboflavin-free conditions (Fig. 3d). Partially, this enhancement of catabolism might be directed to support and fuel the glycolysis. From the other side, the diversity of substrate classes increasingly hydrolyzed during riboflavin starvation suggest an additional role of lysosomes under these conditions, e.g., their role in autophagy. Finally, lysosomal β-galactosidase is considered as a marker of cellular senescence^16^. Although its increase was detected by mass spectrometry measurements (Fig. 3d) and verified by histochemical staining (Fig. 3e), the validity of β-galactosidase to indicate senescence in this experiment setting must be considered cautiously.

In summary, riboflavin starvation of melanoma cells for three days resulted in manifold enhancement of their glycolytic capacity and of lysosomal hydrolysis.

### Riboflavin deficit damages cholesterol synthesis

**Fig. 4:**
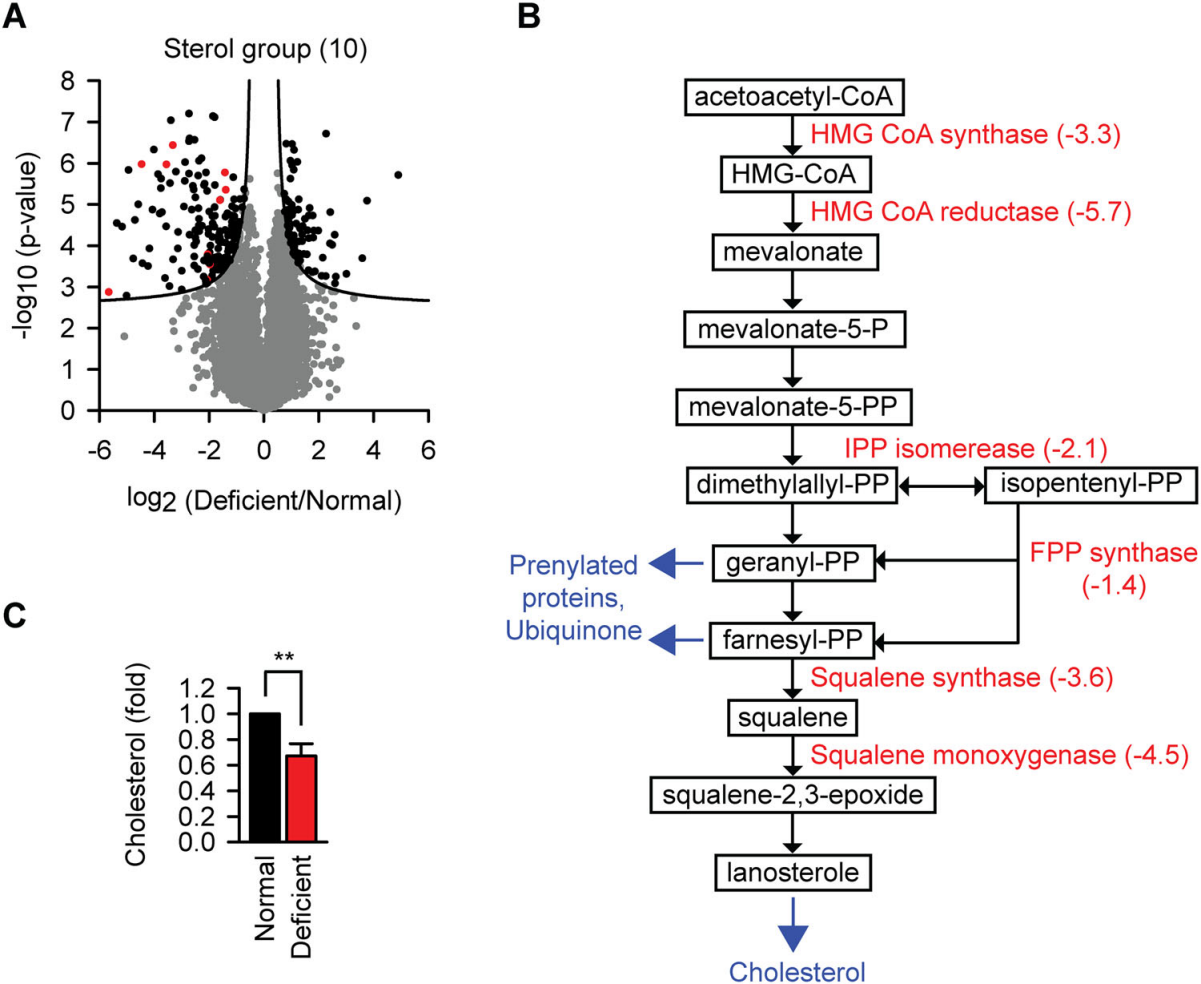
Riboflavin deficit damages cholesterol synthesis. **a** Volcano plot as in Fig. 3b, but with significantly depleted proteins from the sterol biosynthesis group indicated red. **b** Sterol biosynthesis scheme (the mevalonate pathway) with indicated enzymes which were significantly depleted during 3 days culture in riboflavin-deficient medium. The average ratio of downregulation (deficient/normal, log2) is given together with the enzyme name. *N* = 4 independent experiments. **c** Changes of cholesterol per cellular lipid amount upon riboflavin starvation for 3 days. The amount of cholesterol in cells from normal medium was set as 1. **p < 0.01, two-tailed *t* test (*N* = 3, mean ± SD).

Another significantly downregulated functional cluster during riboflavin starvation was related to cholesterol biosynthesis, the mevalonate pathway (Fig. 4a). The observation is surprising because there are only two flavoproteins in this group of enzymes, squalene monooxygenase and delta(24)-sterol reductase (Fig. S4A). Similarly as in the glycolysis upregulation, the downregulation of the mevalonate pathway was extensive, including the key regulator of the pathway HMG-CoA reductase (Fig. 4b). Degradation of HMG-CoA reductase is a well-established negative feedback mechanism in response to cholesterol accumulation^17^. Therefore, we sought to determine cholesterol amount in riboflavin-starved cells to exclude this mechanism as the cause of the observed changes. A remark explaining normalization procedure is needed at this point because we noticed a significant 1.6-fold increase in melanoma cell size upon riboflavin starvation (Fig. S4B). Geometric calculation predicts a 2.5-fold increase of a sphere surface from a 1.6-fold increase of its radius, which has to be considered when comparing membrane lipid composition. Absolute lipid quantification revealed a 2.2-fold higher lipid amount in starved cells (Fig. S4C), the value in line with the calculated expectation and thus used for normalization. The measurements revealed significantly reduced amount of cholesterol in membranes of riboflavin-starved cells (Fig. 4c).

### Membrane lipid composition is altered by riboflavin deficit

**Fig. 5:**
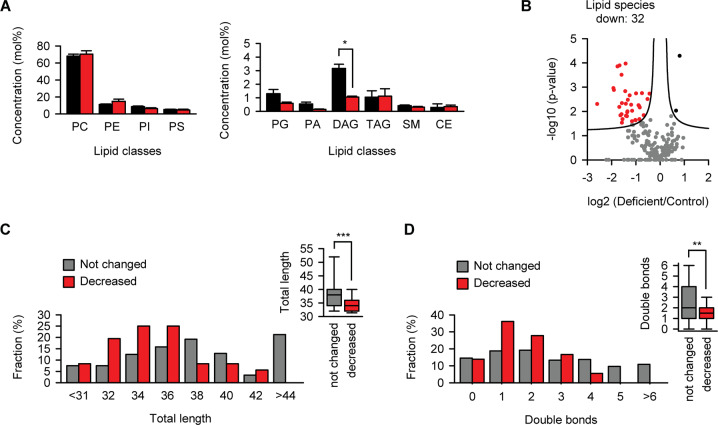
Membrane lipid composition is altered by riboflavin deficit. **a** Effect of riboflavin starvation on cellular lipid classes in melanoma cell line B16. Black, normal medium; red, riboflavin-deficient medium for 3 days. PC, phosphatidylcholine; PE, phosphatidylethanolamine; PI, phosphatidylinositol; PS, phosphatidylserine; PG, phosphatidylglycerol; PA, phosphatidate; DAG, diacylglycerol; TAG, triacylglycerol; SM, sphingomyelin; CE, cholesteryl ester. *p < 0.05, two-tailed *t* test (*N* = 3, mean ± SD). All other differences were not significant. **b** Volcano plot of quantified lipids plotted according to their change in riboflavin-deficient medium for 3 days in B16 cells. The statistical significance of the respective ratios is plotted on the *y*-axis. False discovery rate (FDR) cutoff of 0.05 was used to define the upregulated (black dots) and downregulated (red dots) lipids, which is indicated by continues lines. *N* = 3 independent experiments. **c** Distribution of total length of the fatty acids in the lipid species significantly reduced upon riboflavin starvation compared to that in the unchanged species. Statistical significance was calculated by Mann–Whitney test, ***p < 0.001 (*N* = 3, mean ± SD). **d** Distribution of number of double bonds in fatty acids of the lipid species significantly reduced upon riboflavin starvation compared to that in the unchanged lipid species. Statistical significance was calculated by Mann–Whitney test, **p < 0.01 (*N* = 3, mean ± SD).

The concentration of other lipids was measured by shotgun mass spectrometry (Fig. S5A and Dataset S4). Considering lipid classes, only the concentration of diacylglycerols changed in melanoma cells during riboflavin starvation (Fig. 5a). At the level of individual species, 32 lipids became significantly downregulated during riboflavin starvation (Fig. 5b). Analysis of the aliphatic chain length distribution revealed that the downregulated lipids were significantly shorter (Fig. 5c). Furthermore, analysis of the aliphatic chain saturation revealed that the downregulated lipids were more saturated (Fig. 5d). It remains to be determined what effect the depletion of a small set of short and saturated lipids can have on the fluidity and functionality of cellular membranes during riboflavin starvation. One possibility is that the localization of prenylated proteins at altered membranes is disturbed. In addition, a direct defect of protein prenylation due to the downregulation of the mevalonate pathway can contribute to mislocalization of the respective proteins. To test this possibility, we used EGFP fused with the C-terminal 20 amino acids from HRas known to contain a farnesylation signal (the construct was named EGFP-F). Analysis of EGFP-F revealed no obvious differences in its subcellular distribution in cells cultured in normal or riboflavin-deficient medium (Fig. S5B).

Summarizing the lipidomics analysis we concluded that the acute lack of riboflavin resulted in reduced amount of cholesterol in cells due to the depletion of several key enzymes from the mevalonate pathway and modified lipid composition in membranes of affected cells.

### Riboflavin starvation damages proteins involved in cellular proliferation

**Fig. 6:**
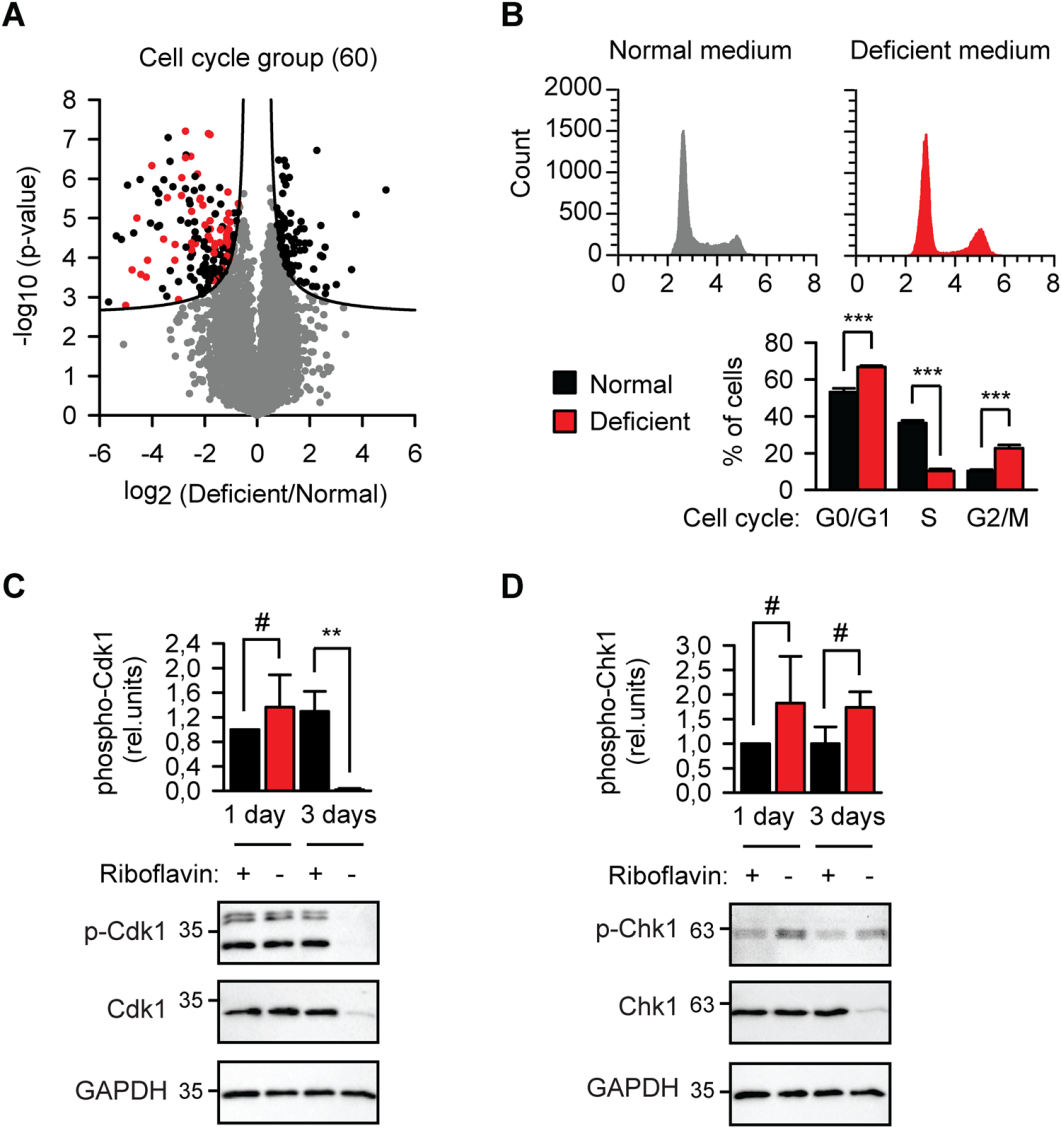
Riboflavin starvation damages proteins involved in cellular proliferation. **a** Volcano plot as in Fig. 3b, but with significantly depleted proteins from cell cycle group indicated red. **b** Cell cycle analysis of B16 cells kept in normal or riboflavin-deficient medium for 3 days. ***p < 0.001, two-tailed *t* test (*N* = 3, mean ± SD). **c** The key cell cycle control kinase Cdk1 is degraded in B16 lysates after 3 days starvation in riboflavin-free medium. Phosphorylation of Cdk1 at Tyr15 was analyzed with phosphospecific antibody (p-Cdk1). GAPDH was used as loading control and for normalization of p-Cdk1 signal. #, not significant difference, **p < 0.01, two-tailed *t* test (*N* = 3, mean ± SD). **d** The DNA damage response kinase Chk1 is degraded in B16 lysates after 3 days starvation in riboflavin-free medium. Phosphorylation of Chk1 at Ser345 was analyzed with phosphospecific antibody (p-Cdh1). GAPDH was used as loading control and for normalization of p-Cdk1 signal. #, not significant difference, two-tailed *t* test (*N* = 3, mean ± SD).

During riboflavin starvation, the largest significantly downregulated functional cluster contained proteins related to cell cycle (Fig. 6a). At least 60 proteins, including the catalytic subunits of DNA polymerases α and δ, PCNA, APC subunits 2 and 7, Aurora kinase A and Polo-like kinase 1, were strongly affected. Consequentially, melanoma cells ceased synthesis of DNA (Fig. S6). Cell cycle analysis found less cells in S phase and more in G0/G1 and G2/M phases (Fig. 6b), which confirmed previous reports of impaired transition through G0/G1^18^ and G2/M^19^. Cyclin-dependent kinase 1 (Cdk1) is the only CDK essential to drive mammalian cell cycle^20^. Since Cdk1 levels are quite constant during cell cycle, its activity is regulated by association with cyclins or inhibited by phosphorylation and association with Cdk inhibitor proteins. We found that riboflavin deficiency led to a profound depletion of Cdk1 which could be confirmed biochemically (Fig. 6c). Earlier, proliferation stop in riboflavin-free medium was proposed to be caused by oxidative DNA damage^21^. Although Chk1 kinase is central for the cellular response to genotoxic stress, we did not observe an absolute accumulation of the active Chk1 as detected with the phosphoSer345-specific antibody (Fig. 6d). On the other side, the protein amount of Chk1 in riboflavin-starved cells dropped substantially, which suggests a very high level of phosphorylation of the remaining kinase (Fig. 6d).

### Riboflavin-starved melanoma cells become vulnerable to alkylating chemotherapy

**Fig. 7:**
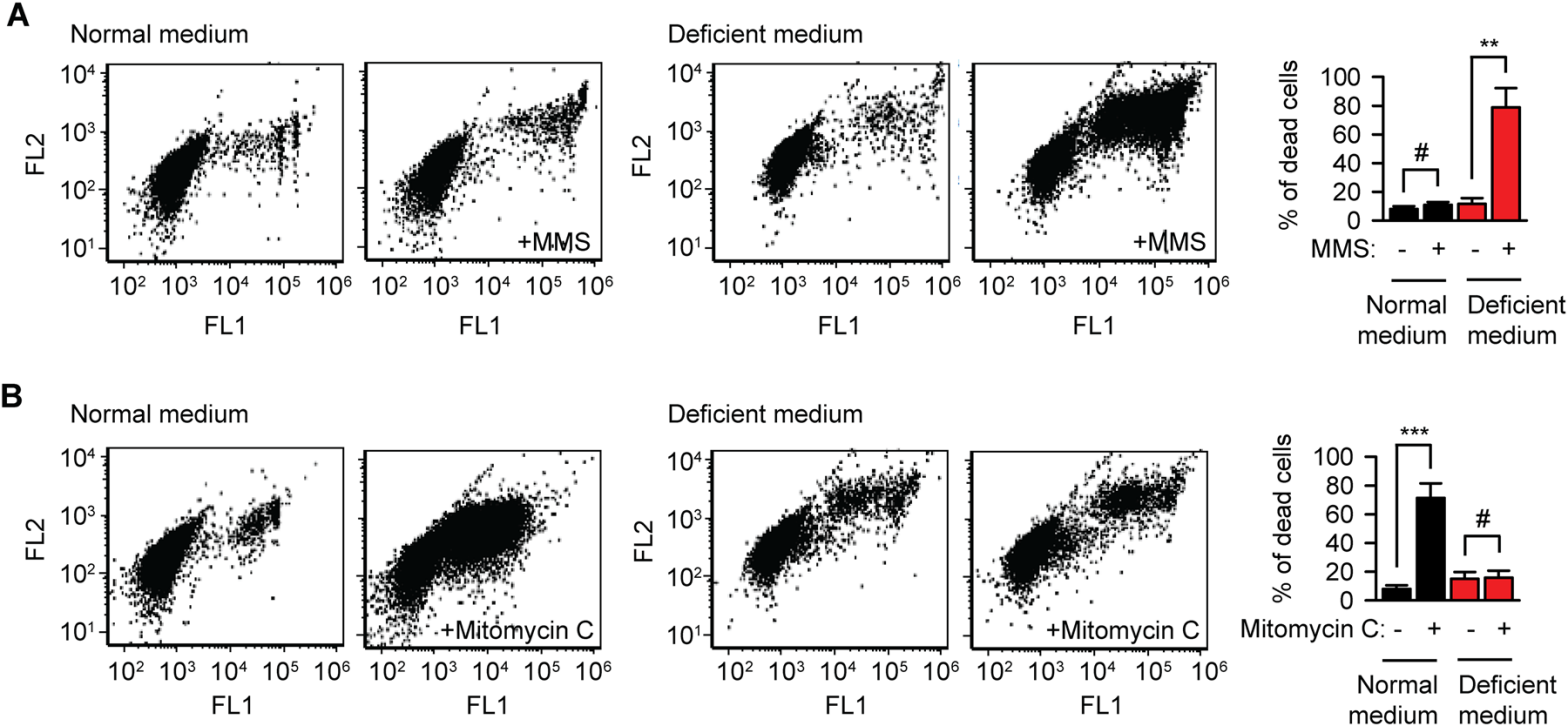
Riboflavin-starved melanoma cells become vulnerable to alkylating chemotherapy. **a** Methyl methanesulfonate (MMS) toxicity in B16 cells kept in normal or riboflavin-deficient medium. After 3 days of starvation, 500 μM MMS were added for additional 24 h and then dead cells quantified using SYTOX dye. ***p < 0.001; #, not significant difference, two-tailed *t* test (*N* = 3, mean ± SD). **b** Mitomycin C toxicity in B16 cells kept in normal or riboflavin-deficient medium. After 3 days of starvation, 100 μM Mitomycin C were added for additional 24 h and then dead cells quantified using SYTOX dye. ***p < 0.001; #, not significant difference, two-tailed *t* test (*N* = 3, mean ± SD).

Alkylating agents are used for chemotherapy of highly proliferative tumors. The targeting specificity is thought to result from the DNA damage of dividing cells^22^. Methyl methanesulfonate (MMS) is the classical experimental reagent that primarily methylates DNA on N7-deoxyguanine and N3-deoxyadenine^23^. Surprisingly, riboflavin-starved non-proliferating melanoma cells became highly vulnerable to the MMS treatment (Fig. 7a). The effect was not restricted to murine melanoma, because human lymphoma cell line Raji became more sensitive to MMS as well (Fig. S7A). MMS does not require metabolic activation^24^, thus the effect cannot be explained by the changed MMS biotranformation under flavin deficiency. As an indirect argument against altered biotransformation, non-proliferating cells in serum-free, but riboflavin-sufficient medium became sensitive to MMS as well (Fig. S7B). The above results suggested that, in addition to DNA, other molecular targets are relevant to explain the increased vulnerability to alkylating agents in non-proliferating cells. In case of riboflavin starvation, membrane alterations due to the inhibition of the mevalonate pathway (Fig. 4b) might contribute to the insufficient anti-apoptotic signaling from cellular membranes (Fig. S7C).

On the other side, Mitomycin C, another alkylating agent, requires reduction to become active (Fig. S7D), and flavoprotein NQO1 was shown to be its key reducing enzyme, especially in tumor tissues^25^. As expected, riboflavin deficiency resulted in a significantly lower potency of the Mitomycin C treatment (Fig. 7b). This result suggests the therapeutic relevance of flavoproteome degradation^12^ and aggregation (this study) and underscores the potential of biochemically informed design of novel chemotherapeutic protocols.

## Discussion

The mechanistic understanding of the three cellular phenotypes of vitamin B2 starvation – reprogramming of energy metabolism, downregulation of the mevalonate pathway and inhibition of DNA-related processes - will be needed in order to proceed with therapeutic implication of riboflavin deficiency.

The key role of flavoproteins in the mitochondrial electron transport chain is well-established^26^. For example, NADH dehydrogenase [ubiquinone] subunit V1 is the core subunit of the the comlex I and contains FMN as cofactor. The subunit of succinate dehydrogenase, the complex II, which is responsible for transferring electrons from succinate to ubiquinone is also a flavoprotein. In addition, at least two flavoproteins, ACAD9 and FOXRED1, are needed to assemble 45 protein into complex I^27^. Not surprisingly, high supplies of vitamin B2 have been reporter to improve clinical symptoms in patients with deficient mitochondrial respiration^28^.

The link between riboflavin supplies and cholesterol biosynthesis is less obvious. Two enzymes in the mevalonate pathway have flavin as their cofactor, squalene monooxygenase and delta(24)-sterol reductase, and their levels dropped significantly during starvation. One possibility is the negative feedback effects of the accumulating substrates of these two flavoproteins on the upstream steps of the pathway. In addition to cholesterol biosynthesis enzymes, LDL receptor, LDL receptor-related protein 6 and Apoliporotein E were downregulated as well (Fig. S4A). These changes could indicate higher-order defects, e.g., disturbance of the endoplasmic reticulum or Golgi apparatus architecture and function. One can predict that systematic analyses of transcriptional, translational and quality control effects on “cholesterol sub-proteome” under vitamin B2 deficiency will turn out very rewarding. Interestingly, statin use has been associated with beneficial effects in cancer patients, which is currently undergoing an accurate and intense epidemiological scrutiny^29^. Statins as inhibitors of the rate-limiting HMG-CoA reduction step in the mevalonate pathway have been suggested to affect several cellular processes with relevance for tumorigenesis, including damage of cell membranes^30^.

Finally, the changes of the DNA synthesis and cell proliferation machinery were most numerous and are highly relevant for tumor therapy. Cell cycle stop under riboflavin starvation was suggested to be due to the oxidative DNA damage^21^. Undoubtedly, flavoproteome defects can lead to the increased generation of reactive oxygen species and the weakening of the cellular anti-oxidative defense. However, only eight proteins related to DNA metabolism were upregulated in contrast to 60 downregulated proteins, which argues against the classical reaction to genotoxic stress (Fig. 6a). What could be the molecular mechanism of such a deep nutritional effect on many DNA-related processes? An inspiring example is represented by the iron–sulfur (Fe–S) cluster biogenesis. MMS19 protein was shown to be required for the cytosolic Fe–S cluster assembly for many DNA metabolism proteins^31,32^. It is conceivable that one or several flavoproteins involved in the Fe–S cluster biogenesis machinery might have similarly numerous consequences if inactivated during riboflavin starvation.

The absence of intracellular riboflavin pools in mammalian cells offers possibilities of controlled restriction of riboflavin supply in combination with other drugs to achieve synthetic lethality. In cases of local tumor growth, regional manipulation of supplies is conceivable, especially as temporal therapy. Available riboflavin analogs must be considered and new substances developed such that efficient competition with vitamin B2 from food or gut microbiome can be ensured^33^. Similarly relevant for the anti-cancer manipulation of vitamin B2 supplies is the recently described riboflavin transporters SLC52A1-3^34^. Their central role in flavin metabolism and flavoproteome function is supported by the pathologies which develop because of transporter mutations and by lethal phenotype of experimental transporter inactivation^35,36^. Our study lays the ground for leveraging the lethality of flavin deficiency as a multiple-target therapeutic approach.

## Supplementary information

Supplemental Information

Figure S1

Figure S2

Figure S3

Figure S4

Figure S5

Figure S6

Figure S7

Dataset S1

Dataset S2

Dataset S3

Dataset S4
