# Supplementary material for: Flavin dependency undermines proteome stability, lipid metabolism and cellular proliferation during vitamin B2 deficiency
Source: Cell Death Dis. 2020 Sep 7;11(9):725. doi: 10.1038/s41419-020-02929-5 (PMC7477094; doi:10.1038/s41419-020-02929-5)
Supplement: Supplementary file 1 — Supplemental Information [file 41419_2020_2929_MOESM1_ESM.docx]

**Supplementary Information**

**Flavin dependency undermines proteome stability, lipid metabolism and cellular proliferation during vitamin B2 deficiency**

Running title: Mechanism of cellular damage by lack of vitamin B2

Adrían Martínez-Limón^1,2,3^, Giulia Calloni^1,2,4^, Robert Ernst^5^, R. Martin Vabulas^1,2,6^

^1^Buchmann Institute for Molecular Life Sciences, Goethe University Frankfurt, Frankfurt am Main, Germany

^2^Institute of Biophysical Chemistry, Goethe University Frankfurt, Frankfurt am Main, Germany

^3^Present address: Institute for Research in Biomedicine (IRB), Barcelona, Spain

^4^Present address: AB SCIEX Germany GmbH, Darmstadt, Germany

^5^Center for Molecular Signaling (PZMS), Institute of Medical Biochemistry and Molecular Biology, Medical Faculty, University of Saarland, Homburg, Germany

^6^Present address: Charité – Universitätsmedizin Berlin, Institute of Biochemistry, Berlin, Germany

Corresponding author: R. Martin Vabulas, Charité – Universitätsmedizin Berlin, Institute of Biochemistry, Charitéplatz 1, 10117 Berlin, Germany. Phone: +49-30-450 528 176, Email: martin.vabulas@charite.de.

**List of Supplementary Information (SI):**

SI Materials and Methods, SI Figure Legends and SI Datasets Legends (1 PDF file)

SI Figures (7 JPG files)

SI Datasets (4 Excel files)

**SI Materials and Methods**

**Reagents, Plasmids and Antibodies**

MG132 was from Enzo Life Sciences (USA); all other chemicals were from Sigma-Aldrich (USA) if not indicated otherwise. 3xFLAG-NQO1 and its mutant P187S were generated as described ^1^. Generation of Ub-EGFP was described earlier ^2^. The following antibodies were used: anti-Akt (Cell Signalling, USA, C67E7; 1:1000), anti-Akt-pT308 (Cell Signalling C31E5E; 1:1000), anti-Akt-pS473 (Cell Signalling D9E; 1:1000), anti-AMPK (Cell Signalling D5A2; 1:1000), anti-AMPK-pT172 (Cell Signalling 40H9; 1:1000), anti-BrdU Alexa Fluor 488 (Merck, Germany, FCMAB101A4; 1:200), anti-Chk1 (Cell Signalling 2G1D5; 1:1000), anti-Chk1-pS345 (Cell Signalling 133D3; 1:500), anti-FLAG (Sigma-Aldrich F1804; 1:1000), anti-GAPDH (Cell Signalling 14C10; 1:1000), anti-GFP (Roche 11814460001; 1:1000), and anti-HSP70 (StressGen, USA, SPA-810; 1:1000).

**Blue-Native Gel Electrophoresis**

B16 cells were transfected with 15 µg of 3xFLAG-NQO1 mammalian expression construct by means of electroporation and plated on 10-cm dish. After 6 hours, media was changed and cells were incubated for 24 hours either in normal or riboflavin-deficient medium. Cells were then treated with 10 µM MG132 O/N. Next morning cells were harvested by trypsinization, resuspended in native lysis buffer (50 mM Bis-Tris HCl pH 7.2, 5 mM NaCl, 10% glycerol, 2 mM MgCl_2_, 0.1% Triton X-100, and protease inhibitor cocktail from Sigma-Aldrich) and incubated on ice for 15 min. 100 U benzonase (Merck Millipore) were added and lysates were incubated for additional 30 min at room temperature. After centrifuging samples for 20 min at 17.000 g, supernatant was normalized to contain 1.5 µg/µL protein. Coomassie Blue G-250 stain (Carl-Roth, Germany) was added to each sample to a final concentration of 0.025%. NativePAGE™ 4-16% Bis-Tris precast gels (Invitrogen, USA) were used to run 20 µg of native sample per lane. Running buffer (50 mM Bis-Tris HCl pH 6.8, and 50 mM Tricine) with 0.02% Coomassie Blue G-250 was used during the first running phase (ca. half of the gel) at 150 V at 4ºC. Second half was run using 0.002% Coomassie Blue G-250 at 250 V until the blue dye front reached the bottom of the gel. Native gel electrophoresis was used to detect high molecular weight NQO1 aggregates, thus the entire part of gels above the NQO1 band is shown in figures. Because aggregates do not migrate according to their size, molecular weight markers were omitted from these analyses. Before transfer, gel was soaked in 2x Transfer buffer (5 mM Bis-Tris HCl pH 7.2, 50 mM Bicine, and 2 mM EDTA) for 10 min at room temperature. Proteins from native gel were transferred onto PVDF membrane for 70 min at 180 V and at 4ºC using 1x Transfer buffer. PVDF membrane was washed with 100% methanol for 3 min at room temperature to remove the excess of the stain and incubated in 8% acetic acid for 15 min at room temperature. Immunoblotting protocol was continued as described above. To solubilize the NQO1 aggregates, supernatants after centrifugation were incubated with 10 µM FAD or riboflavin for 5 min at room temperature where indicated.

**Preparation of Pull-Downs for Proteomics Analysis**

2 x 10^6^ B16 cells were plated in the evening on 10-cm dishes. Next morning, cells were transfected with 10 µg of 3xFLAG-NQO1 wild-type and P187S mutant eukaryotic expression vectors using polyethylenimine (PEI) (DNA:PEI ratio 1:3 using a 1 mg/mL PEI solution). 6 h later, cells were washed extensively with PBS and normal or riboflavin-deficient medium was addded. After 24 h, 5 µM MG132 was applied and starvation was continued overnight. Cells were collected after trypsinization, washed twice with PBS, resuspended in ice-cold lysis buffer (50 mM Tris HCl pH 7.4, 150 mM CaCl_2_, 1 mM EDTA pH 8.0, and 0.1% IGEPAL), kept on ice for 15 min and then sonicated for 3 sec at 50% output with a MS72 sonotrode. Cell lysates were pre-cleared by centrifugation at 17.000 x g for 15 min at 4ºC. In the meantime, anti-FLAG M2 Affinity Gel (Sigma-Aldrich) was prepared by washing resin with PBS twice and once with lysis buffer. Absolute protein concentration in samples was normalized to 2 µg/µL. 250 µL of the normalized lysate (500 µg protein) were mixed with the affinity resin and incubated for 3 h at 4 ºC while rotating. After incubation, the resin was washed five times with TBS (50 mM Tris HCl pH 7.5, and 150 mM NaCl) and transferred to protein low binding tubes (Eppendorf, Germany). One final wash with mass spectrometry buffer (20 mM Tric HCl pH 7.4, and 150 mM NaCl) was performed.

**Lipidomics Analysis**

B16 cells were incubated for 3 days under normal or riboflavin-deficient conditions. 1.5x10^6^ cells were harvested by trypsinization and washed twice with PBS. After the final wash, PBS was carefully removed, cell pellets were frozen in liquid nitrogen and kept at -80ºC until analysis. Lipid quantification by shotgun mass spectrometry was performed by Lipotype GmbH (Dresden, Germany) as detailed below. Lipids were extracted using chloroform and methanol. Samples were spiked with lipid class-specific internal standards prior to extraction. After drying and re-suspending in MS acquisition mixture, lipid extracts were subjected to mass spectrometry using a hybrid quadrupole/Orbitrap mass spectrometer equipped with an automated nano-flow electrospray ion source in both positive and negative ion mode.

Lipid identification using LipotypeXplorer ^3^ was performed on unprocessed mass spectra. For MS-only mode, lipid identification was based on the molecular masses of the intact molecules. MSMS mode included the collision-induced fragmentation of lipid molecules and lipid identification was based on both the intact masses and the masses of the fragments. Prior to normalization and further statistical analysis, lipid identifications were filtered according to mass accuracy, occupation threshold, noise and background. Lists of identified lipids and their intensities were stored in a database optimized for the particular structure inherent to lipidomic datasets. Intensity of lipid class-specific internal standards was used for lipid quantification.

Bioinformatic data analysis was performed using Perseus (version 1.5.2.6) ^4^. Mol% values for the identified lipid species in any of 3 independent experiments were imported in Perseus as expression columns and log2 transformed. Control and riboflavin deficient samples were grouped and the lipid species significantly changed upon riboflavin starvation were identified by two-sample t-test at a permutation-based FDR cutoff of 0.05 and s0 = 0.1.

**Cholesterol Measurement**

B16 cells were cultured for 3 days under riboflavin-deficient conditions. A total of 1.5x10^6^ cells were counted, washed twice with PBS and re-suspended in 50 µl distilled water. Lipids were extracted as described ^5^. All the steps were performed at room temperature. 270 µl methanol solution with 3% acetic acid was added to the cell suspension, followed by the addition of 1 ml methyl-*tert*-butyl ether. Samples were incubated for 1h while rotating and phase-separation was then induced by adding 250 µl distilled water. After further incubation for 5 min, samples were centrifuged for 10 min at 15 000g. Approximately 800 µl of the upper organic phase was collected using a glass Pasteur pipette. 400 μl of reextraction solution (20 ml methyl-*tert*-butyl ether, 6 ml methanol with 3% acetic acid, 5 ml water) was added to the remaining polar fractions to induce a second round of separation. Samples were incubated for 20 min under vigorous shaking at 1300 rpm and centrifuged again for 10 min at 15.000 g. The upper phase (ca. 450 µl) was collected and pooled with the fraction from the first extraction. Solvent was completely evaporated with speed vacuum machine. The pellet obtained was re-suspended in 60 µl methanol and directly used for cholesterol measurement using Amplex Red Cholesterol kit Assay (Thermo Fisher Scientific). All reactions were carried out in reaction buffer (100 mM KH_2_PO_4_ pH 7.4, 50 mM NaCl, 5 mM cholic acid, and 0.1% Triton X-100) in a total volume of 100 µl in 96-well black plates. Cholesterol standard curve was prepared with concentrations: 0, 50, 100, 200, 400, and 800 ng/ml. Every reaction contained 150 µM Amplex Red, 1 U/ml horseradish peroxidase, 1 U/ml cholesterol oxidase, and 0.1 U/ml cholesterol esterase. Reactions were incubated for 30 min at 37ºC and fluorescence signal was measured using 560 nm excitation and 590 nm emission. Blank signal (reaction buffer with no cholesterol) was subtracted from all values obtained. Fluorescence measurements from three different sample dilutions were obtained for each experiment. Values of starved samples were divided by 2.2 to correct for the increase of total lipid amount per cell.

**Mass Spectrometry of NQO1 Interactors**

*Sample preparation.* Pulled down proteins were processed on-beads for LC-MS/MS analysis as following. Beads were re-suspended in 50 μl 8M urea/50 mM Tris HCl pH 8.5, reduced with 10 mM DTT for 30 min and alkylated with 40 mM chloroacetamide for 20 min at 22°C. Urea was diluted to a final concentration of 2 M with 25 mM Tris HCl pH 8.5, 10% acetonitrile and proteins were digested with trypsin/lysC mix (mass spec grade, Promega, USA) overnight at 24°C. Acidified peptides (0.1% trifluoroacetic acid) were desalted and fractionated on combined C18/SCX stage tips (3 fractions). Peptides were dried and resolved in 1% acetonitrile, 0.1% formic acid.

*LC-MS/MS.* LC-MS/MS was performed on a Q Exactive Plus equipped with an ultra-high pressure liquid chromatography unit (Easy-nLC1000) and a Nanospray Flex Ion-Source (all three from Thermo Fisher Scientific). Peptides were separated on an in-house packed column (12.5 cm length, 75 µm inner diameter) with 2.4 µm Reprosil-Pur C18 resin (Dr. Maisch GmbH, Germany) using a gradient from mobile phase A (4% acetonitrile, 0.1% formic acid) to 30% mobile phase B (80% acetonitrile, 0.1% formic acid) for 60 min followed by a second step to 60% B for 30 min, with a flow rate of 300 nl/min. MS data were recorded in data-dependent mode selecting the 10 most abundant precursor ions for HCD with a normalized collision energy of 27. The full MS scan range was set from 350 to 2000 m/z with a resolution of 70000. Ions with a charge ≥2 were selected for MS/MS scan with a resolution of 17500 and an isolation window of 1.6 m/z. The maximum ion injection time for the survey scan and the MS/MS scans was 80 ms, and the ion target values were set to 3x10^6^ and 10^5^, respectively. Dynamic exclusion of selected ions was set to 60 s. Data were acquired using Xcalibur software (Thermo Fisher Scientific).

*Data analysis with MaxQuant*. MS raw files from 4 biological replicates were analyzed with MaxQuant (version 1.5.3.30) ^6^ using default parameters. Enzyme specificity was set to trypsin and lysC and a maximum of 2 missed cleavages were allowed. A minimal peptide length of 7 amino acids was required. Carbamidomethylcysteine was set as a fixed modification, while N-terminal acetylation and methionine oxidation were set as variable modifications. The spectra were searched against the UniProtKB mouse FASTA database (downloaded in January 2016, 50189 entries) for protein identification with a false discovery rate of 1%. Unidentified features were matched between runs in a time window of 2 min. In the case of identified peptides that were shared between two or more proteins, these were combined and reported in a protein group. Hits in three categories (false positives, only identified by site, and known contaminants) were excluded from further analysis. For label-free quantification (LFQ), the minimum ratio count was set to 1. The proteomics data have been deposited to the ProteomeXchange Consortium via the PRIDE partner repository (https://www.ebi.ac.uk/pride/archive/) with the data set identifier PXD013312.

*Data analysis with Perseus.* Bioinformatic data analysis was performed using Perseus (version 1.5.2.6). Proteins identified in the pulldowns were included in the analysis if they were quantified in all biological replicates in at least one experimental condition. Missing LFQ values were imputed on the basis of normal distribution with a width of 0.3 and a downshift of 1.8. Proteins enriched in the pulldown over background binding for each condition were identified by two-sample t-test at a p-value cutoff of 0.05. The “PD/background” in the respective plots stands for the ratio of quantified protein from NQO1 vector-transfected cells versus the same protein from empty vector-transfected cells.

**MS Data of Flavoproteome changes**

To analyze flavoproteome changes upon riboflavin starvation for 3 days in B16 murina melanoma cells, we used the published data set ^1^. Identified proteins were filtered to include in the analysis only those quantified in at least 3 of 4 biological replicates in each group (control and riboflavin deficient condition). Missing LFQ values were imputed on the basis of normal distribution with default parameters. Proteins significantly changed upon riboflavin starvation were selected by two-sample t-test with FDR < 0.001 and s0 = 0.1. Categorical annotations were added in Perseus and a Fisher’s exact test with a FDR threshold of 0.02 was run for GO term enrichment analysis.

**Statistical Analyses**

All repetitions in this study were independent biological repetitions performed at least three times if not specified differently. To identify significantly increased proteins in pulldowns in mass spectrometry analyses, a two-sample t-test analysis of grouped biological replicates was performed using a FDR cutoff of 0.001 with s_0_ = 0.1 (for proteome changes) or a p-value cutoff of 0.05 (for pulldowns). Categorical annotation was added in Perseus and a Fisher exact test with a p-value threshold of 0.02 was run for GOslim term enrichment analysis. Statistical significance of the median difference for non-normally distributed data was calculated by Mann-Whitney test. Means and standard deviations were calculated from at least three independent experiments.

1. Martínez-Limón, A. *et al.* Recognition of enzymes lacking bound cofactor by protein quality control. *Proc. Natl. Acad. Sci. U.S.A.* **113**, 12156–12161 (2016).

2. Vabulas, R. M. & Hartl, F. U. Protein synthesis upon acute nutrient restriction relies on proteasome function. *Science* **310**, 1960–1963 (2005).

3. Herzog, R. *et al.* A novel informatics concept for high-throughput shotgun lipidomics based on the molecular fragmentation query language. *Genome Biol.* **12**, R8 (2011).

4. Tyanova, S. *et al.* The Perseus computational platform for comprehensive analysis of (prote)omics data. *Nat. Methods* **13**, 731–740 (2016).

5. Eggers, L. F. & Schwudke, D. Shotgun Lipidomics Approach for Clinical Samples. *Methods Mol. Biol.* **1730**, 163–174 (2018).

6. Cox, J. & Mann, M. MaxQuant enables high peptide identification rates, individualized p.p.b.-range mass accuracies and proteome-wide protein quantification. *Nat. Biotechnol.* **26**, 1367–1372 (2008).

**SI Figure Legends**

**Figure S1. Depletion of Riboflavin Leads to Coaggregation of Apoprotein NQO1 with Bystander Proteins in Melanoma Cells.**

(**A**) Lysates of NQO1-transfected cells were prepared and aggregation was analyzed as described in Fig. 1A for MG-132 containing samples. To solubilize aggregates *in vitro*, 10 μM FAD or riboflavin were added for 5 min. The total amount of NQO1 was determined by western blotting after SDS-PAGE (middle gel). GAPDH was used as loading control. #, not significant difference; ***p<0.001, two-tailed t test (N = 3, mean ± SD).

(**B**) Overlap of wild-type (WT) and mutant (MUT) NQO1 interactors in normal and riboflavin-deficient medium.

**Figure S2. Proteome Damage during Riboflavin Starvation does not Affect Translation.**

(**A**) Overlap of wild-type apo-NQO1 interactors (WT) with the interactors of mutant NQO1 in normal (MUT in gray) and riboflavin-deficient (MUT in red) medium.

(**B**) Translation rate is similar in B16 cells after 2 days in normal or riboflavin-deficient medium. Accumulation of Ub-EGFP protein upon proteasome inhibition for 5 h was compared by means of lysate western blotting. GAPDH was used as loading control. #, not significant difference, two-tailed t test (n = 3, mean ± SD).

**Figure S3. Riboflavin Starvation Results in Metabolic Reprogramming of Melanoma Cells.**

(**A**) ATP levels in B16 cells were measured after 3 d in riboflavin-free medium and are represented as fraction of ATP in cells kept in normal medium. **p<0.01, two-tailed t-test (N=4, mean ± SD).

(**B**) Significantly enriched GO Biological Process categories among proteins depleted in B16 cells after 3 d in riboflavin-free medium. The categories are grouped into three classes.

(**C**) Significantly enriched GO Biological Process categories among proteins upregulated in B16 cells after 3 d in riboflavin-free medium.

**Figure S4. Riboflavin Deficit Damages Cholesterol Synthesis.**

(**A**) Proteins involved in mevalonate pathway, which were significantly (FDR<0.001) depleted in B16 cells after 3 d in riboflavin-free medium. The log2 values of the respective ratios are listed. *, significant changes with FDR<0.01.

(**B**) B16 cell size increase upon riboflavin starvation for 3 days as measured by flow cytometry. Left panel, cell size distribution of cells in normal medium (black) and riboflavin-deficient medium (red). Right panel, comparison of cell size median in different conditions. *p<0.05, two-tailed t test (N = 3, mean ± SD).

(**C**) Absolute lipid amount in 1.8x10^6^ B16 cells quantified by mass spectrometry as a sum of the analyzed lipid listed in Fig. 3D. *p<0.05, two-tailed t test (N = 3, mean ± SD).

**Figure S5. Membrane Lipid Analysis during Riboflavin Deficiency.**

(**A**) Color-coded riboflavin starvation-induced changes as log10(deficient/normal), red and blue, as an increase and decrease of individual lipid species, respectively. CE, cholesteryl ester; DAG, diacylglycerol; PA, phosphatidate; PC, phosphatidylcholine; PE, phosphatidylethanolamine; PG, phosphatidylglycerol; PI, phosphatidylinositol; PS, phosphatidylserine; SM, sphingomyelin; TAG, triacylglycerol.

(**B**) Cellular distribution of farnesylated EGFP in B16 cells upon 3 d incubation in normal and riboflavin-deficient medium. Representative pictures of one from three independent experiments are shown. Scale bar, 50 μm.

**Figure S6. Riboflavin Starvation Affects DNA Synthesis.**

BrdU incorporation in DNA of B16 cells kept for 3 d in riboflavin-deficient medium is inhibited. 10 mM BrdU pulse was performed for 5 h. DAPI staining was used to visualize cell nuclei. DIC, differential interference contrast. Scale bar, 20 μm.

**Figure S7. Analysis of the Chemotherapeutic Vulnerability of Riboflavin-starved Cells.**

(**A**) Methyl methanesulfonate (MMS) toxicity in Raji cells kept in normal or riboflavin-deficient medium. After 3 days of starvation, 500 μM MMS were added for additional 24 h and then dead cells quantified using SYTOX dye. **p<0.01, two-tailed t test (N = 3, mean ± SD).

(**B**) MMS toxicity in B16 cells kept in serum-containing or serum-free medium for 1 day. 500 μM MMS was added for additional 24 h and then dead cells quantified using SYTOX dye. **p<0.01, two-tailed t test (N = 3, mean ± SD).

(**C**) Retention of active Akt is affected under riboflavin deficiency. Kinetics of Akt activation upon addition of 500 μM MMS was analyzed using phospho-Thr308-specific (pT308) and phospho-Ser473-specific (pS473) antibodies in B16 lysates after 3 d culture in normal or riboflavin-free medium. GAPDH and total Akt were used as loading controls. #, significant difference, **p<0.01, ***p<0.001, two-tailed t test (N = 3, mean ± SD).

(**D**) Activation scheme of Mitomycin C by the NQO1-catalyzed intracellular reduction.

**SI Dataset Legends**

**Table S1 (Excel file). MaxLFQ Quantitative Data and Identifiers of Wild-Type and Mutant NQO1 Interactors.** This file contains label-free quantification of proteome changes under specified conditions. Mass spectrometry files were processed with MaxQuant as details in *SI Materials and Methods*.

**Table S2 (Excel file). Wild-type NQO1 Interactors.** This file contains the list of WT NQO1 interactors pulled down under normal and riboflavin-deficient conditions. The average enrichments over the background control (AVG LFQ PD/BG) from four biological replicates are listed.

**Table S3 (Excel file). Mutant NQO1 Interactors.** This file contains the list of mutant NQO1 interactors pulled down under normal and riboflavin-deficient conditions. The average enrichments over the background control (AVG LFQ PD/BG) from four biological replicates are listed.

**Table S4 (Excel file).** **Lipidomics Analysis of B16 Cells Cultured in Normal and Riboflavin-Deficient Medium.** This file contains the relative quantification of the lipid species identified under normal conditions and after 3 days of riboflavin starvation. Lipid quantification is reported as mol % of the total lipid content.
